# Institutional review boards - a mixed blessing

**DOI:** 10.1186/1755-7682-4-19

**Published:** 2011-06-20

**Authors:** Taimur Saleem, Umair Khalid

**Affiliations:** 1 Medical College, Aga Khan University, Stadium Road, Karachi 74800, Pakistan

## Abstract

Institutional Review Boards (IRBs) are an important checkpoint for all types of research in medicine. Although these bodies originated primarily in the developed world, they have special contemporary consideration in the context of developing countries due to the large number of clinical trials being conducted in these regions with the financial support of large pharmaceutical companies. IRBs are vital to ensure that all scientific investigation is conducted in a manner that is transparent, scientifically feasible and ethically sound. However, they have also been variably criticized for introducing unnecessary and often protracted bureaucracy and red tape into the system. There is a need to reorganize and better delineate the exact functions of the IRBs in view of the dynamic changes in the realm of research so that they can function in a more efficient, judicious and effective fashion.

## Background

Institutional Review Boards (IRBs) are "the officially empowered guardians of the rights and welfare of human research subjects" [1]. These bodies have gradually evolved to become essential check points for all types of research in medicine. They function as the "final arbiter of scientific inquiry" [2] and play a central role in the approval, evaluation, review and monitoring of research [3].

## Historical perspective

The conception of IRBs can be attributed to the process of peer-review in scientific literature [1] and primarily originated in the developed world. The two events that most significantly underlined the need for having agencies such as the IRB to oversee research activities involving human participants are the Nuremberg Doctor's Trial and Tuskegee Syphilis Study. The former occurred in the aftermath of World War II whereby Nazi scientists were tried for war crimes involving human experimentation. Consequently, the Nuremberg Code was formulated in 1947 by the Nuremberg Military Tribunal as a 10-point statement. The most salient feature of this code was that 'all participation in research must be voluntary' [4]. In the Tuskegee Syphilis Study, about 400 African American patients diagnosed with syphilis were included. The aim of this study was to observe the natural course of the disease. However, these patients were not given effective treatment for syphilis, even though effective therapies for syphilis had been discovered by that time.

These events led to the formulation of multiple documents that have championed the cause of research subjects and research ethics. These include the Declaration of Helsinki in 1966, Dr. Beecher's essay 'Ethics and Clinical Research' in 1966, the National Research Act in 1974, the Vancouver Group's recommendations, the landmark Belmont Report in 1979, the Common Rule in 1981, the Food and Drug Administration (FDA) regulations in 1980 and 1981 in the United States (U.S.), the Council for International Organizations of Medical Sciences' (CIOMS) manual 'International Ethical Guidelines for Biomedical Research involving Human Subjects' in 1982, the Guideline for Good Clinical Practice (GCP) in 1996 and the National Bioethics Advisory Commission's (NBAC) report in 1998 [4,5]. A detailed analysis of these documents reveals that although there is a general consensus over the rudimentary principles of ethical research, there are many areas where such concurrence is lacking [4].

## Merits of IRBs

There is an ever increasing scope of research in the contemporary era. Myriad innovations in medicine need to be investigated in a systematic fashion before their mainstream implementation [1]. Authors are required by journals to comply with the guidelines of their respective IRBs and to obtain IRB approval before embarking on any scientific venture. These guidelines are usually based on institutional and national recommendations as well as World Medical Association Declaration of Helsinki Statement of Ethical Principles for Medical Research [3].

Several instances in recent years have strengthened the case and need for approval of research studies by regulatory research bodies such as IRBs. In 2007, the Nigerian government and local government of Kano filed two separate lawsuits against a large pharmaceutical company for $7 billion and $2 billion respectively. The company was sued on multiple accounts including failure to obtain parental consent and federal approval before giving the experimental antibiotic trovafloxacin to 100 children with meningococcal meningitis in Kano; additional allegations were leveled against the company for use of an antibiotic that caused deafness, paralysis, brain damage, blindness, and death in children [6]. Interestingly, the FDA had not approved the drug in question for use in children. In adults, the drug was approved for use in 1997, but withdrawn in 1999 and currently is restricted for use only in life-threatening infections [6].

The importance of IRBs has also been highlighted by the misconduct of some researchers. There are reports that have implicated investigators at highly reputed academic centers in the U.S. for conducting experimental research in other regions of the world such as India and China without the knowledge or consent of their university or government or without a proper ethics review of their research protocol [6].

IRBs are thus vital to ensure that all scientific investigation is conducted in a manner that is transparent, scientifically feasible and ethically sound. Subjecting study protocols to stringent rules and regulations also provides a potential shield against litigation for the institution where the research is being conducted [2].

Another merit of IRBs is that the subjects are also potentially protected from interference by funding bodies and sponsoring agencies [1]. Sponsorships from industrial giants indeed make it possible to conduct large scale, multicenter trials. However, such sponsorships can potentially give these agencies leverage to manipulate how a trial is conducted with a possibility of comprise in scientific integrity. This can include irregularities in randomization of subjects, testing of unsafe therapies, "tweaked" or inaccurately performed statistical analysis, delayed, modified or no reporting of adverse events and overall outcomes. The presence of IRBs ensures that scientific integrity is upheld throughout the process so that the sponsors do not wield undue influence over the trial. In addition, the privacy and confidentiality of the subjects is protected.

## Pitfalls of IRBs

However, despite being championed as the "moral police" of the district of scientific research, IRBs have also been criticized for a number of reasons. Quite simply, IRBs are thought to introduce redundant friction in the system as they often "go over-board" with the execution of tasks entrusted to them [2]. They represent an intrusion of bureaucracy and excessive red tape into medicine to the extent of being "frustrating", "consternating" and "paternalistic" for the researchers [2,7]. Most of the procedures and processes of the IRB are perceived as being extraneous or "extra-regulatory" and the body itself is often overloaded and understaffed to handle the ever burgeoning volume of research presented to it [1].

The decisions made by IRBs are often absolute and there is little opportunity for investigators to appeal against their decisions [2]. These decisions maybe perceived by investigators as not based on logic, "bizarre" [2] or stemming from a clash of egos. Further deterrence against IRB scrutiny may stem from fears of losing important research funding due to the protracted trajectory involved [6]. The stringent requirements of IRB approval by an increasing number of medical journals also presents an impediment for independent practitioners and private or nonacademic health centers [3,7].

It should be acknowledged that "guidelines merely guide...they do not bind" [6]. IRBs are potentially open to interpret and modify consensus guidelines for conducting research according to loco-regional subtexts; this introduces an element of variability into the whole equation [8]. Alternatively, this very facet can be considered a strength of the IRB system as it leaves a margin of allowance for the choice of the means used to achieve a common set of goals [1,5]; an appraisal of the axiom that 'no two fingerprints are alike'. Interpretations may diverge because of differently nuanced personalities, national trends and even elected personnel in government [1]. Subtle conflicts may also exist between the IRBs if the study is a multi-center one, as is often the case with large randomized controlled trials. Although necessary, gaining approval separately from the IRBs at each of the participating institutions can considerably tax the financial resources in addition to prolonging the whole process by several months. Also, researchers wishing to introduce any change, however small, in the research protocol have to apply for a re-approval of the protocol with the new changes from their respective IRBs [2]. On-going research must also be continually reviewed by the IRB to detect any safety issues or ethical breaches etc.

IRBs generally would not have the discretion to withhold approval of a safe study even if it is not lucrative to the university. However, in view of the great financial recession in the current decade, there could be a conflict of interest leading to the possibility that IRBs may be inclined to approve the most feasible, lucrative and perhaps potentially unsafe studies from investigators who are bringing the most funding for the institution [1]. This "capitalistic" mindset can undermine the foundation and vision of scientific inquiry in a highly erosive manner.

IRBs also utilize considerable human work force and financial resources at academic institutions. In a survey of 488 IRBs, the average IRB committee was found to consist of 14 members [9]. In multiple surveys [10,11], it has been reported that the medial cost incurred by IRBs ranges between $76,000 to $770,000 per annum (range $76,000 - $1.15 million). This roughly translates into $300 to $800 per action. Some detractors of IRBs do not believe that these bodies, as they are in ineffective existence today, merit such a high level of spending. One point that was subsequently raised was that these studies only focused on academic medical centers, whereas clinical trials are also conducted by contract research organizations which may be more efficient as far as the utilization of resources and human capital is concerned [12].

At academic medical centers, faculty members either volunteer or are assigned to contribute their time and expertise to IRBs [12]. In addition to the significant time commitment the review of research protocols entails, this represents an added responsibility beyond what these faculty members are usually remunerated for. This may cause a decreased level of motivation among the members of the IRB and ultimately translate into a sub-optimal quality of the review process.

## Issues in developing countries

A special mention needs to be made of the complexities arising due to research being conducted in developing countries. These societies are entrenched in a complex labyrinth of socio-economic and cultural intricacies, including a phenomenal veneration for physicians, which needs to be navigated by the researchers. International research is extremely important in balancing out inequities in research between the developed and developing world [13] as less than 10% of global resources are being spent on 90% of the world's health issues [14]. However, much hue and cry has also been raised in recent years over the growing number of international drug companies who are funding major clinical trials in the developing world. This change in paradigm is due to a number of possible reasons including lesser costs, less red tape, illiterate populace and deficient or malleable ethical frameworks and monitoring systems in the developing world; these elements provide a highly fertile ground for companies to "outsource" trials to the developing countries [15].

In addition to approval from ethical review committees in developing countries, ethical approval for such projects is also required from IRBs in the developed countries where these pharmaceutical giants are usually based. However, there is no guarantee that both the participants and the researchers in the developing world are fully cognizant of the implications such approval entails. Much of the paperwork involved, including lengthy consent forms, may be formulated in jargon that is too complex or altogether unfamiliar for or inapplicable to the populace in developing countries [16].

In international research done at sites in developing countries, new therapies are tested against existing therapies, placebos or "normal standards of care". However, there is growing debate over the definition of "normal, fair and reasonable standard of care" in the developing world. In particular, placebo controlled trials involving HIV patients came under heavy fire for being unethical as they did not allegedly compare newer therapy against the current best standard of care [17]. Supporters of such trials have, however, adopted a different stance on the subject. They argue that in developing countries, a placebo can be considered as a "prevailing standard of care" because in the poverty-stricken third world, the standard of care is often no treatment at all [18]. At the other extreme, the assumption that the standards of care in the developed world are the "universal standards of care" is also flawed [17]. While these may be the most advanced treatments available, impulsively labeling them as the best treatments for the developing world is not correct, particularly if the argument is not rationalized in a context-specific manner. Serious consideration needs to be given to the severe resource constraints in developing countries where advanced and often expensive treatment can only be afforded by a handful of people belonging to the elite strata of society. The debate on placebo controlled trials is ultimately a complex one. It should be noted that for some diseases, the FDA may consider placebo as an acceptable control in a trial [19]. Therefore, the standard of care in the developing countries in most instances, if not all, should not be placebo or 'no treatment at all'; it should be 'treatment for all' - a treatment modality that is accessible, affordable and morally and medically justifiable. Also, future debate on "standards of care" needs to consider additional aspects such as structure and efficiency of the national health system [20]. The structure influences provision of different levels of care to patients at different locations while system efficiency influences the quality of care available in a particular location. Consequently, a "national standard of care" is not synonymous with a "local standard of care" [20].

At the end of the day, the fundamental question remains. Are IRBs in the developing world more or less effective as compared to their counterparts in the developed world? The answer probably lies in a gray zone rather than being written in explicitly black and white ink. Undoubtedly, subjects in developing countries require IRBs to be their 'guardian angels' by virtue of their unique circumstances and socio-demographics. However, IRBs in the developing world are plagued by problems such as inconsistency, scarcity, inexperience and lack of funds [6]. The latter may place research subjects in developing world in a vulnerable position. Anecdotal evidence exists that local ethics review committees do not possess sufficient influence to modify protocols given by sponsors from the developed world [21].

## Solutions

We may consider monetary reimbursement for faculty members serving on the IRBs to add incentive to this part of their jobs. More recently, there is a shift towards reimbursing the members of the IRB for their effort and time [22]. However, whether this approach will work or not is debatable as often the primary goal and motivation behind working in academia is to garner academic merit and credit [23] rather than pursuit of monetary benefits.

In the current situation, most of the energy and time of IRBs is expended in "beating about the bush" - in activities that are low-yield, protracted, non-standardized and seemingly impractical [11,23] that may have led IRBs to sink into "mission creep" [24]. Excessive focus on procedures, documentations and definitions only serves to fulfill perfunctory aspects of research [24]. In a study assessing IRB processes across 68 U.S. hospitals for a single multicenter study, it was seen that about one-third of the IRBs required the principal investigator to be from the same institution and about 27% required evidence of human subjects research training [11]. Many IRBs require multiple copies of the application from the investigators; still many may not accept an electronic version of the application. Upto eight copies of the IRB application were requested at some centers in one study while less than half of the IRBs had forms available on their website [11]. Investigators should be allowed to submit IRB application and all associated materials online to save time and resources. Also, redundancy in the application materials should be avoided as they constitute low-yield procedures that only increase the review time and the researcher's frustration without practically improving subject safety [11]. They also often distract the IRBs from performing more important functions. In a survey of 488 IRBs, only 58% had a written policy regarding research integrity - a core dimension of IRB functioning [9]. Lackey very aptly commented in one of his papers that, "in moral matters, as in practical life, it is more important to worry about avoiding disasters than achieving perfection" [18].

One solution to the IRB problem is to make multiple IRBs at the same institution to deal with research of different types in order to distribute the workload, better utilize expertise and expedite review timelines [12]. However, this needs to be reconciled with the fact that increased functional costs will be incurred from such administrative "splitting" and "IRB specialization".

Alternatively, the solution could lie in unifying IRBs at different academic centers into a "single, cooperative academic IRB". The benefits derived from this unification would include decreased overall functioning costs, increased efficiency, and removal of redundancy or duplication of review necessitated by having IRBs at every academic center especially for multicenter trials, ultimately making the whole process more streamlined [12]. A third solution could be outsourcing the reviews to "independent" or "commercial" IRBs [16]. In the latter two scenarios, it would be important to negotiate the balance of power, authority and control between the local institution and non-institutional IRB [22].

Perhaps and more importantly, it may be worthwhile in the long-term to invest in "where the rubber meets the road" - training of the investigators both in sound scientific methodology and ethics of research [22]. The powers that be can churn out one guideline after the other but they are all fruitless if the morality and ethical code of researchers with regards to their research subjects is flawed to begin with [15].

For research done in developing countries, it is important for IRBs in the developed world to safeguard the rights of the research subjects in the developing countries while respecting the wisdom, experience and advice from IRBs in the developing countries. The drug companies funding the clinical trials must not be allowed to exploit the weaker ethical systems in the developing countries [15]. Profitability must not blind-sight the regard for the safety of participants. Shortcuts for fulfilling ethical imperatives in developing world should be avoided [21]. IRBs in the developing world need to be fully cognizant of the emerging challenges of international research in developing countries. Being important stakeholders in the process, they must participate in the formulation of principles governing the ethics of international research. Local empowerment and engendering sustainable capacity in bioethical study and expertise is also a very important step in that direction [13]. We quote the words of Abdallah Daar here, "So long as all the ethicists are in the North, and the South is just the recipient of ethical principles, nothing will change!'' [13,25].

The complete umbrella of IRB functioning also needs to be evaluated and defined. IRB functions have gradually expanded to include verification of conflicts of interest, health record confidentiality and waiver of regulations etc. However, IRBs were not originally designed, intended or funded to support these additional functions [24]; as a consequence IRBs are overworked, overloaded and understaffed because their functions have mushroomed without a parallel modification in structure to buttress these added responsibilities.

## Conclusions

IRBs exert considerable influence over the morale of researchers and the research milieu of an institution [26]. Discourse continues on whether the modern IRB is part of the problem or the solution. Are these IRBs the counterproductive white elephants of science or the indispensible white knights for legal and ethical imperatives in research? Are IRBs the malevolent adversaries or the honest well-wishers of researchers? [27]

Two interesting paradoxes exist here: first, IRBs police researchers about the application of evidence-based methods to conduct research whereas their own procedures have considerable variability due to lack of such relevant data for their own practices and considerations [28] and secondly, "a committee is only as ethical as the members who constitute its body" [15]. IRBs are often constituted by the colleagues of the investigators who themselves have conducted research at some point as well. Ultimately, it is perhaps most appropriate to describe IRBs as a mixed blessing! Research, especially involving human subjects, is subject to rigorous regulations in the current medical landscape with good reason. However, there is a need to take account of the special administrative and logistic challenges faced by investigators due to strict red tape from IRBs.

At the end of the day, we acknowledge that scientific inquiry needs to entail appropriate safeguards to the research subjects. Some researchers, but not all, will have common scientific sense with an intact moral compass. But all doors don't have the same key just as one glove doesn't fit all; the profile of the genuinely well-intentioned researcher, therefore, can't be generalized in an indiscriminate manner. Martinson et al showed in 2005 that one-third of all scientists in the U.S. had been involved in research misconduct at some point in the past 3 years [29]. IRBs exist to provide a much needed check and balance in the system. However, at the same time, all research needs to be effectively executed and efficiently planned to economize resources [30] and IRBs need to be restructured to better achieve this aim in the most judicious manner possible [2]. It may better suit the IRB to be responsive, not reactive, to the changes in today's research culture [1].

## Competing interests

The authors declare that they have no competing interests.

## Authors' contributions

TS was involved in the conception, literature search, writing and critical revision of the manuscript. UK was involved in the literature search and drafting the manuscript. All authors have read and approved the final version of the manuscript.
